# Long‐term outcome of immunologic autograft engineering

**DOI:** 10.1002/jha2.404

**Published:** 2022-02-24

**Authors:** Luis F. Porrata, David J. Inwards, Stephen M. Ansell, Ivana N. Micallef, Patrick B. Johnston, Jose C. Villasboas, Jonas Paludo, Svetomir N. Markovic

**Affiliations:** ^1^ Division of Hematology Department of Medicine Mayo Clinic Rochester Minnesota USA; ^2^ Department of Medical Oncology Mayo Clinic Rochester Minnesota USA

**Keywords:** autograft absolute lymphocyte count, autologous peripheral blood hematopoietic stem cell transplantation, survival

## Abstract

Our phase III trial reported that autograft‐absolute lymphocyte count (A‐ALC) improved survival post‐autologous peripheral blood hematopoietic stem cell transplantation (APBHSCT) for a short‐term follow‐up of 2 years. We evaluated retrospectively in our phase III trial patients that the A‐ALC still confers survival benefit with a longer follow‐up. With a median follow‐up of 127.6 months, patients infused with an A‐ALC ≥ 0.5 × 10^9^ cells/kg experienced better overall survival (HR = 0.392, 95% confidence of interval [CI]: 0.224–0.687, *p* < 0.001) and progression‐free survival (HR = 0.413, 95% CI: 0.253–0.677), *p* < 0.0004). This study supports that A‐ALC provides long‐term survival benefit post APBHSCT.

## 1 INTRODUCTION

A limitation of our phase III study (NCT00566228) that showed autograft‐absolute lymphocyte count (A‐ALC) improves survival post‐autologous peripheral blood hematopoietic stem cell transplantation (APBHSCT) is the short‐term follow‐up of 2 years [1]. To assess if the A‐ALC still provides improved clinical outcomes, we evaluated the survival prognostic ability of the A‐ALC and autograft natural killer cells (A‐NK) in a post‐hoc analysis in the patients enrolled in our phase III trial with a longer term follow‐up.

## 2 METHODS

### 2.1 Patient cohort

This study was approved by the Mayo Clinic Institutional Review Board according to the regulation of the Declaration of Helsinki. Sixty‐two patients were accrued to the modified setting arm and 60 patients to the standard setting arm from December 10, 2007 until October 12, 2010. A total of 56 patients in the modified setting arm and 55 in the standard setting arm were able to finish the trial. These 111 patients were studied in this retrospective study. All patients baseline characteristics has been previously published [1]. The primary end point of the study was to investigate if the A‐ALC affects overall survival (OS) and progression‐free survival (PFS) in longer follow‐up in lymphoma patients treated with APBHSCT. OS was defined as the date of autograft infusion to the date of death due to any cause. PFS was defined as the time from the date of autograft infusion to disease progression or death due to any cause.

### 2.2 Statistical analysis

OS and PFS were analyzed using the approach of Kaplan and Meier [2]. Differences between survival curves were tested for statistical significance using the two‐tailed log‐rank test. Univariate and multivariate analysis was performed using the Cox proportional hazard model [3]. Variables with a *p*‐value < 0.2 in the univariate analysis were included in the multivariate analysis. An A‐ALC ≥ 0.5 × 10^9^ cells/kg cut‐off value tested was based on our previous publication [1]. The cut‐off choice value for autograft natural killer cells (A‐NK) to assess survival was based on the utility as a marker for the clinically relevant binary outcome of death/survival using the receiver operating characteristics curves (ROC) and area under the curve (AUC). A *K*‐fold cross‐validation with *K* values of 10 was performed to validate the A‐NK cut‐off obtained by the ROC and AUC curves. For the autograft lymphocytes subset analysis, patients autograft samples for each apheresis collection were collected and studied by flow cytometry as previously published [1]. Chi‐square tests and Fisher exact tests were used to determine relationships between categorical variable as appropriate. The Wilcoxon rank test was used to determine associations between continuous variables and categorical and nonparametric tests were used to evaluate associations for continuous variables. All *p*‐values represented were two‐sided and statistical significance was declared at *p* < 0.05.

## 3 RESULTS

The median age at the time of ABPHSCT was 57 years (range: 20–74). The median follow‐up for the living patients (N = 52) was 127.6 months (range: 5.9–158.1 months). The transplant‐related mortality at day 100 was 3.6% (4/111). Forty‐four patients had died due to lymphoma; 4 patients due to myocardial infarction; 3 patients due to therapy‐related acute myelogenous leukemia; 2 patients due to septic shock; 2 patients due to pneumonia; 1 patient of heart failure; 1 patient of prostate cancer; 1 patient due to acute respiratory distress syndrome; and 1 patient of anaplastic astrocytoma.

**FIGURE 1:**
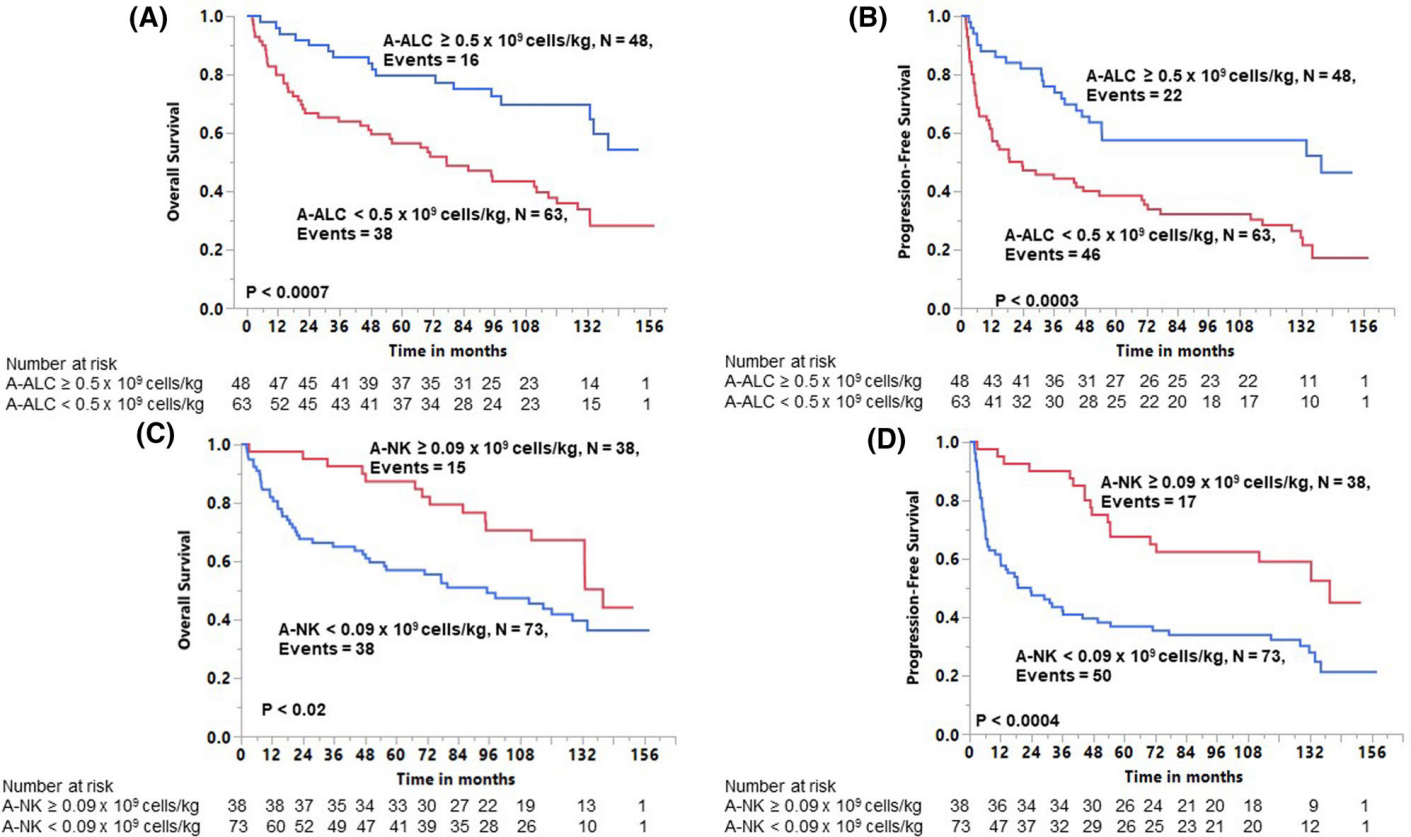
**(A)** Overall survival based on the infused autograft absolute lymphocyte count (A‐ALC). **(B)** Progression‐free survival based on the infused autograft absolute lymphocyte count. **(C)** Overall survival based on the infused autograft natural killer cells (A‐NK). **(D)** Progression‐free survival based on the infused autograft absolute lymphocyte count

The OS and PFS were observed to be superior for patients infused with an A‐ALC ≥ 0.5 × 10^9^ cells/kg. The 13‐year OS rates for the A‐ALC ≥ 0.5 × 10^9^ cells/kg group was 54% (95% confidence interval [CI], 36–72%) and for the A‐ALC < 0.5 × 10^9^ cells/kg group was 28% (95% CI, 18–42%) (*p* < 0.0007) (Figure 1A). The 13‐year PFS rates for the A‐ALC ≥ 0.5 × 10^9^ cells/kg group was 46% (95% CI, 30–64%) and for the A‐ALC < 0.5 × 10^9^ cells/kg group was 17% (95% CI, 6–32%) (*p* < 0.0003) (Figure 1B).

**Supporting information** **includes the univariate analysis table with the variables tested for OS and PFS**. Of the original tested lymphocyte subsets, A‐NK as a continuous variable was an OS and PFS predictor. Using the ROC and AUC curves with internal validation of the AUC curves from the *k*‐fold cross‐validation with *k = *10, the best cut‐off value for the A‐NK was 0.09 × 10^9^ cells/kg with an AUC of 0.7, *p* < 0.03, and sensitivity of 0.73 and specificity of 0.66.

The 13‐year OS rates for the A‐NK ≥ 0.09 × 10^9^ cells/kg group was 44% (95% CI, 25–65%) and for the A‐NK < 0.09 × 10^9^ cells/kg group was 36% (95% CI, 25–49%) (*p* < 0.02) (Figure 1C). The 13‐year PFS rates for the A‐NK ≥ 0.09 × 10^9^ cells/kg group was 45% (95% CI, 26–65%) and for the A‐NK < 0.09 × 10^9^ cells/kg group was 21% (95% CI, 12–35%) (*p* < 0.0004) (Figure 1D).

Table 1 shows that in multivariate analysis both A‐ALC and A‐NK were independent predictors for OS and PFS.

**TABLE 1:** Multivariate analysis for overall survival and progression‐free survival including all the patients

	Overall survival			Progression‐free survival		
Variables	HR	95% CI	*p*	HR	95% CI	*p*
A‐ALC ≥ 0.5 × 10^9^ cells/kg	0.367	0.195–0.690	<0.001	0.477	0.271‐0.840	<0.01
A‐NK ≥ 0.09 × 10^9^ cells/kg	0.411	0.275–0.892	<0.01	0.490	0.266‐0.903	<0.02
Age, years ≤ 60	0.681	0.316–1.465	0.3			
CD34 × 10^6^ cells/kg (continuous variable)	0.115	0.014–1.672	0.2	0.178	0.028‐1.080	0.07
Complete response prior to transplant	0.538	0.303–0.951	<0.03	0.486	0.292‐0.808	<0.005
Extranodal disease < 2	0.673	0.431–1.331	0.4	0.393	0.156‐0.993	<0.05
LDH (U/L) normal	0.554	0.259–1.186	0.1	0.960	0.488‐1.886	0.9
IPI index < 3	0.698	0.268–1.818	0.4	0.738	0.345‐1.578	0.4
Stage I/II vs. III/IV	0.858	0.330–2.232	0.7	0.634	0.257‐1.565	0.3

Abbreviations: A‐ALC, Autograft absolute lymphocyte count; A‐NK, autograft natural killer cell; IPI, International Prognostic Index; LDH, Lactate dehydrogenase (normal value < 222 U/L).

## 4 DISCUSSION

The current study shows superior survival for lymphoma patients that received an A‐ALC ≥ 0.5 × 10^9^ cells/kg for longer follow‐up after APBHSCT. Our group recently published a 3‐year follow‐up matched‐control study comparing clinical outcomes before and after we changed our clinical practice collecting A‐ALC ≥ 0.5 × 10^9^ cells/kg in conjunction with CD34 stem cells on April 1, 2017 showing better survival of patients infused with an A‐ALC ≥ 0.5 × 10^9^ cells/kg compared with those infused with an A‐ALC < 0.05 × 10^9^ cells/kg [4]. A‐ALC was an independent predictor for OS and PFS in the match‐control study as well as this present study; thus, providing more clinical evidence of the A‐ALC as a survival biomarker in APBHSCT. A‐NK as a subset of the A‐ALC was also an independent predictor for survival in the study. In the allogeneic setting, the NK immunoglobulin‐like receptors (KIRs) affect survival [5]. Our group reported that the infusion of autograft NKp30 NK cells (activating receptor) and KIR2DL2 NK cells (inhibitory receptor) impact clinical outcomes post‐APBHSCT [6]. In multiple myeloma, patients post‐APBHSCT showing evidence of the adaptive NKG2C NK cells (activating receptor) expansion showed decreased relapse rates [7]. These findings argue that the KIR mechanism of tumor targeting seen in the allogeneic stem cell transplantation might also apply in the APBHSCT. Recently, our group published a more detailed analysis of the autograft collected and infused immune effector cells affecting clinical outcomes post‐APBHSCT [6] and our findings have recently confirmed [8, 9, 10]; thus, supporting the concept of autologous graft versus tumor effect [11]. This current study with a longer term follow‐up supports our practice change to collect A‐ALC ≥ 0.5 × 10^9^ cells/kg in addition to CD34 to improve clinical outcomes for lymphoma patients undergoing APBHSCT.

## AUTHORSHIP CONTRIBUTIONS

Conception and design: LFP;

Data collection and analysis: LFP and SNM;

Data interpretation: all authors;

Manuscript writing: LFP;

Manuscript editing: all authors;

Final approval of the manuscript: all authors.

## CONFLICT OF INTEREST

The authors declare no conflict of interest.

## Supporting information

Supporting information
