# Supplementary material for: Long‐term outcome of immunologic autograft engineering
Source: EJHaem. 2022 Feb 24;3(2):488–91. doi: 10.1002/jha2.404 (PMC9176079; doi:10.1002/jha2.404)
Supplement: Supplementary file 1 — Supporting information [file JHA2-3-488-s001.docx]

**Manuscript ID: eJhaem-2022-01-0363**

**Supplemental section**

**Revision 1**

**Porrata et al. Long-term outcome of immunologic autograft engineering**

**Supplemental Table**

**Table 1. Univariate analysis for overall survival and progression-free survival**

|  | Overall Survival | Progression-Free Survival |
| --- | --- | --- |
| Variables | HR 95%CI P | HR 95%CI P |
| A-ALC ≥ 0.5 x10^9^ cells/kg | 0.392 0.224-0.687 <0.001 | 0.413 0.253-0.677 <0.0004 |
| A-CD3 x 10^9^ cells/kg (continuous variable) | 1.898 0.592-5.127 0.3 | 1.643 0.541-4.254 0.3 |
| A-CD4 x 10^9^ cells/kg (continuous variable) | 1.852 0.161-6.492 0.6 | 2.049 0.432-8.623 0.4 |
| A-CD8 x 10^9^ cells/kg (continuous variable) | 1.860 0.406-6.652 0.4 | 1.843 0.409-6.610 0.4 |
| A-NK x 10^9^ cells/kg (continuous variable) | 0.308 0.095-0.889 <0.003 | 0.248 0.087-0.652 <0.007 |
| A-NK ≥ 0.09 x 10^9^ cells/kg | 0.518 0.292-0.919 <0.02 | 0.393 0.230-0.670 <0.0006 |
| Age, years ≤ 60 | 0.544 0.318-0.930 <0.03 | 0.921 0.661-1.378 0.7 |
| **Aggressive vs indolent histology** | **1.485 0.842-2.620 0.2** | **1.177 0.723-1.916 0.5** |
| CD34 x 10^6^ cells/kg (continuous variable) | 0.788 0.676-0.919 <0.02 | 0.174 0.040-0.753 <0.02 |
| Complete response prior to transplant | 0.552 0.323-0.943 <0.02 | 0.463 0.284-0.753 <0.002 |
| Extra-nodal disease < 2 | 0.251 0.112-0.563 <0.0008 | 0.340 0.154-0.752 <0.008 |
| IPI index < 3 | 0.379 0.221-0.650 <0.0004 | 0.557 0.335-0.924 <0.02 |
| LDH (U/L) normal | 0.441 0.255-0.761 <0.003 | 0.682 0.422-1.104 0.1 |
| Performance status <2 | 0.700 0.316-1.551 0.4 | 0.788 0.376-1.650 0.5 |
| Stage I/II vs III/IV | 0.535 0.230-1.252 0.1 | 0.377 0.163-0.875 <0.02 |

**Abbreviations: A-ALC = Autograft absolute lymphocyte count; A-CD3 = autograft CD3; A-CD4 = autograft CD8; A-NK = autograft natural killer cell; IPI = International Prognostic Index; and LDH = Lactate dehydrogenase (abnormal value ≤ 222 (U/L)).**
